# Supplementary material for: Control of pili synthesis and putrescine homeostasis in Escherichia coli
Source: eLife. 2025 Apr 3;13:RP102439. doi: 10.7554/eLife.102439 (PMC11968103; doi:10.7554/eLife.102439)
Supplement: Supplementary file 1. — A regression line was calculated and the correlation between each set of transcriptomes was noted on the graph. Higher R2 values indicate greater similarity between the transcriptomes. [file elife-102439-supp1.pdf]

Comparision of the logCPM of the *speB* mutant with and without 1.0mM putrescine

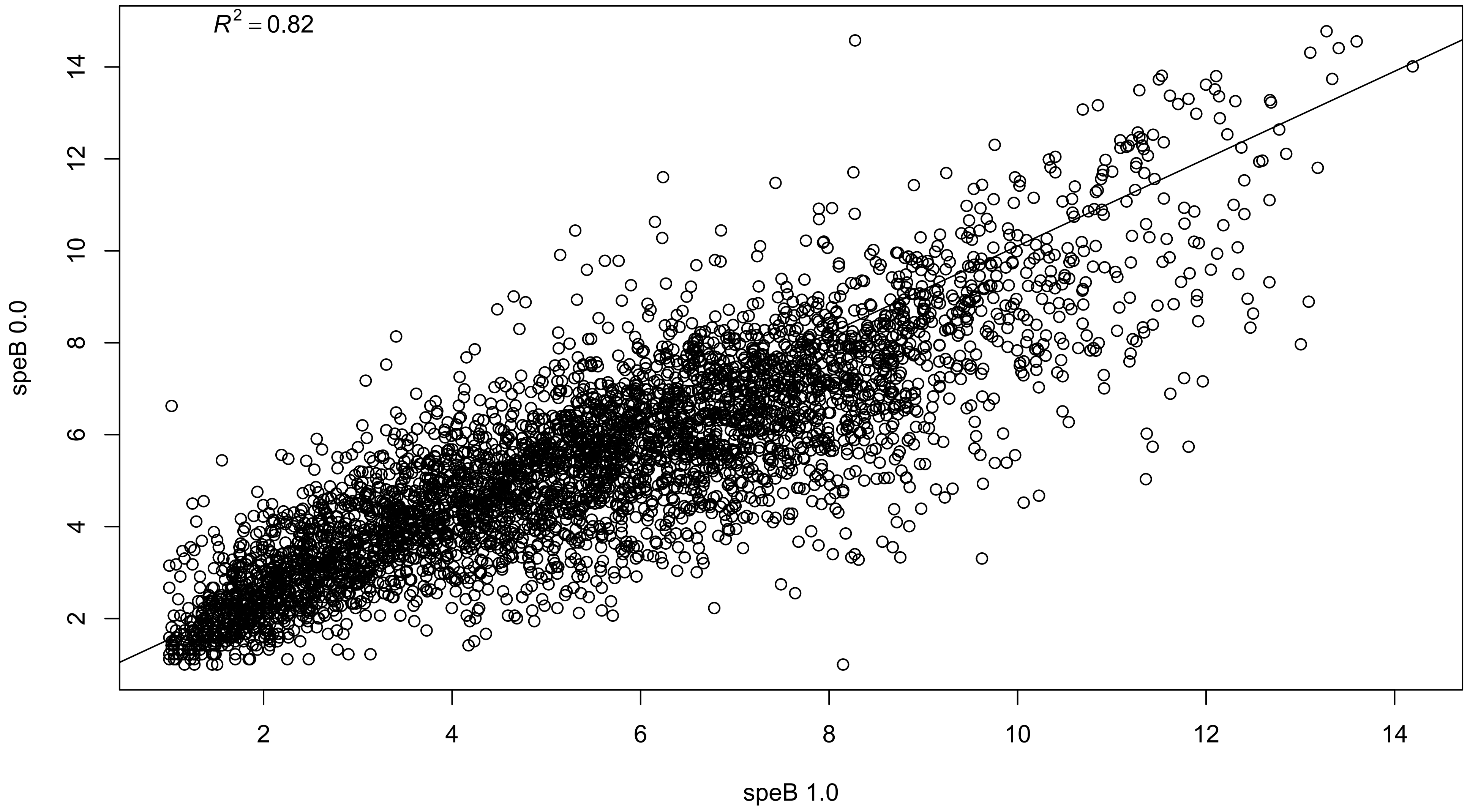

Comparision of the logCPM of W3110 and a speB mutant without putrescine

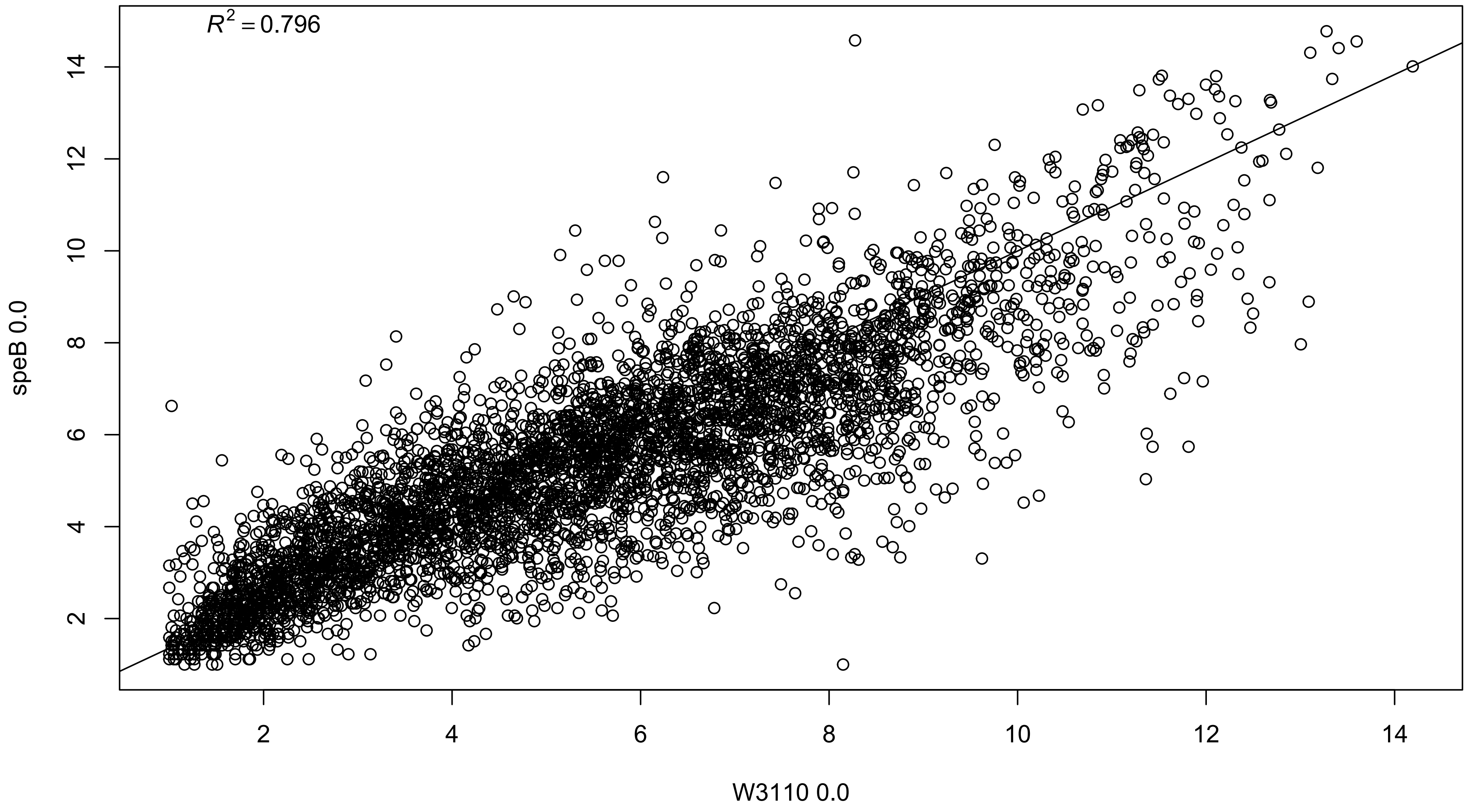

Comparision of the logCPM of W3110 with 1.0mM putrescine and a speB mutant without putrescine

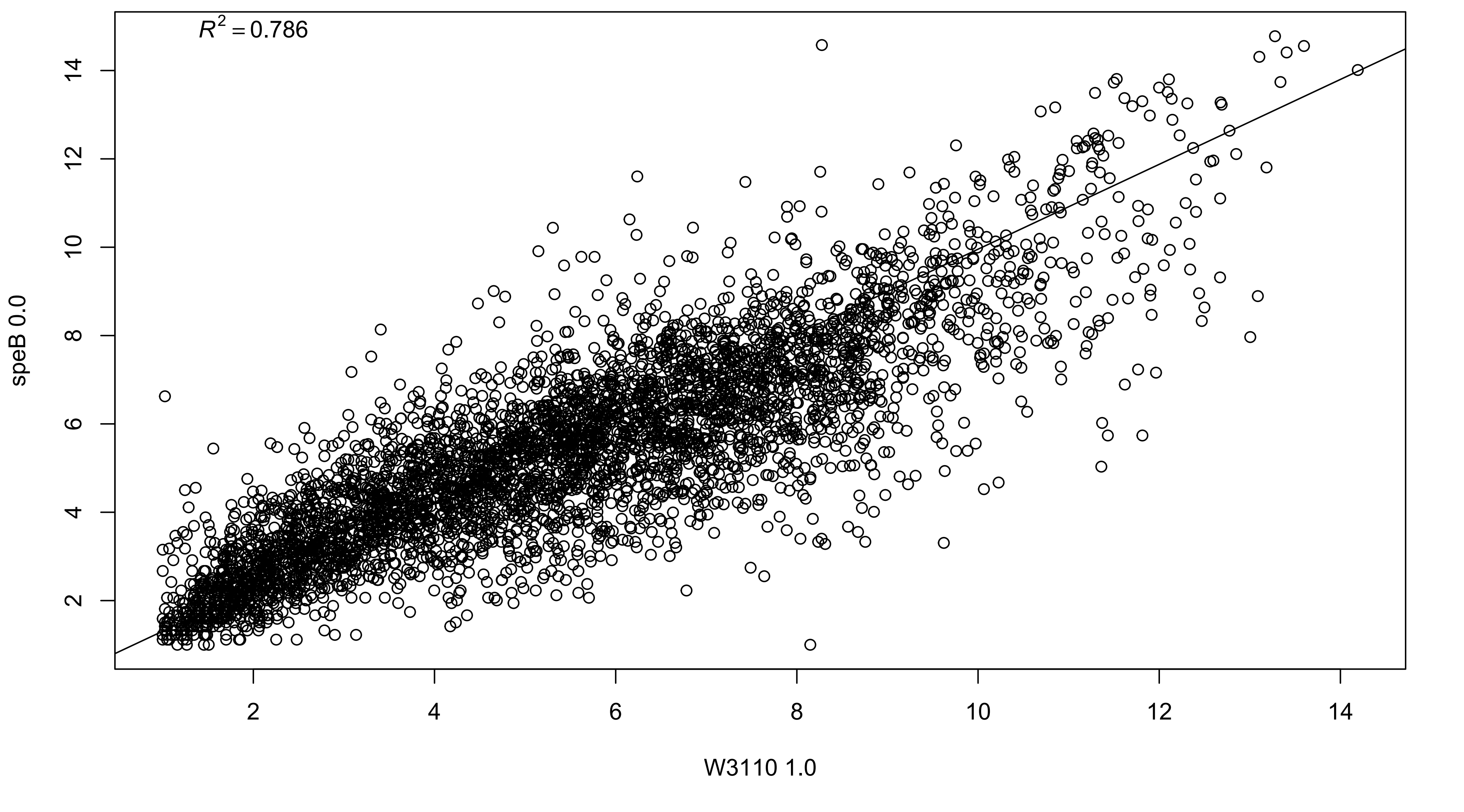

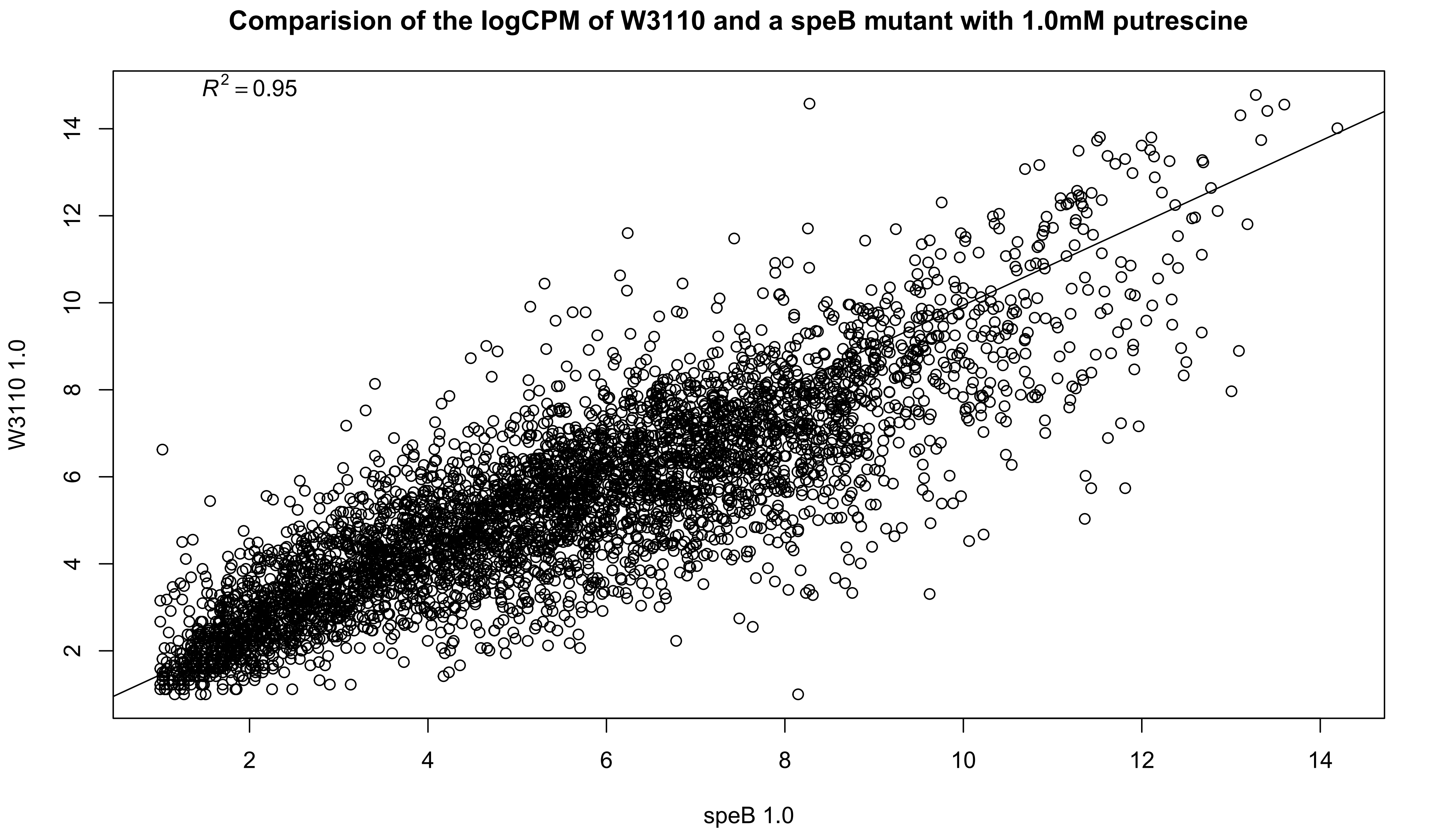
Comparision of the logCPM of W3110 and a speB mutant with 1.0mM putrescine
$R^2 = 0.95$
W3110 1.0
speB 1.0
2
4
6
8
10
12
14

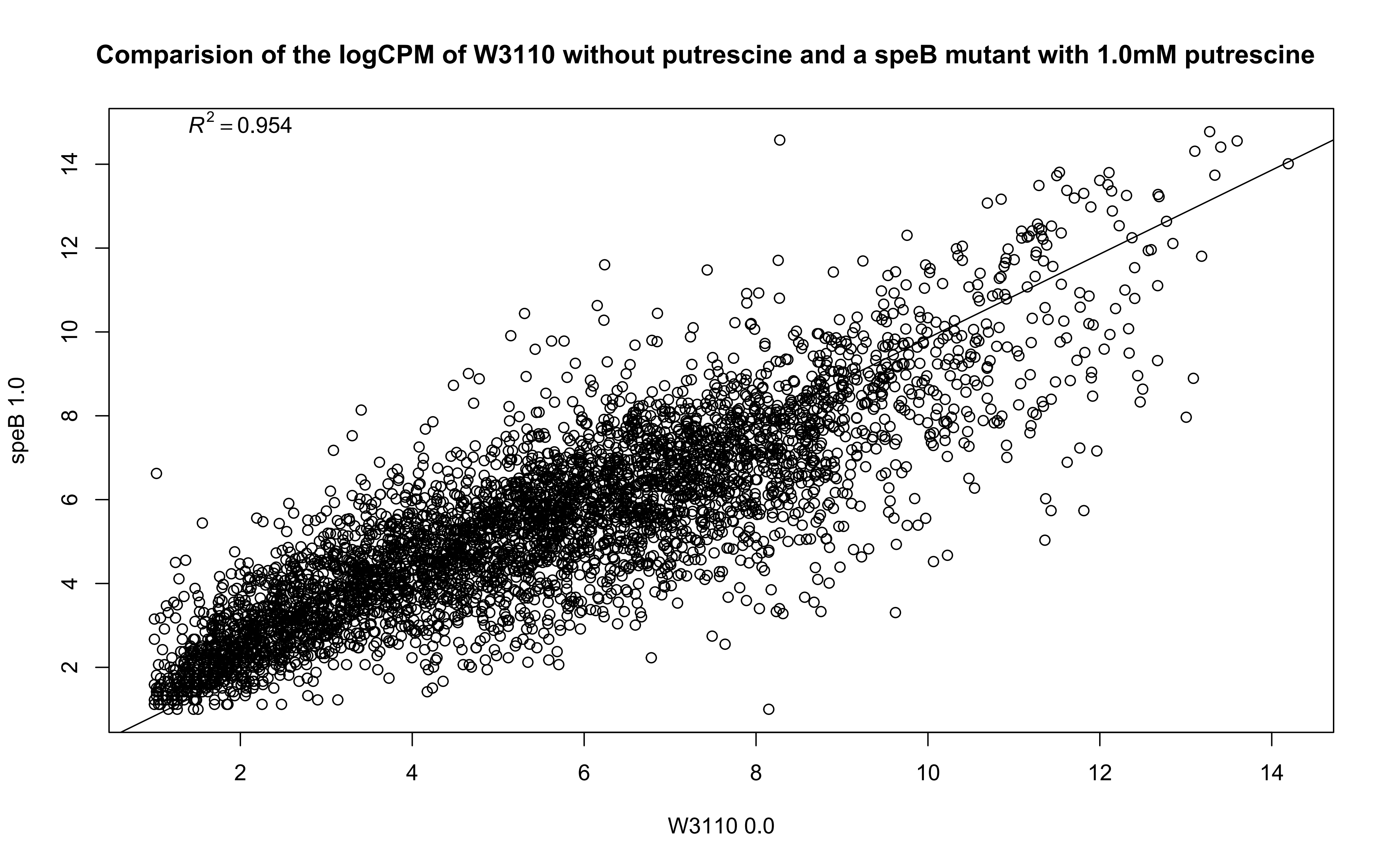
Comparision of the logCPM of W3110 without putrescine and a speB mutant with 1.0mM putrescine
$R^2 = 0.954$
speB 1.0
W3110 0.0
2
4
6
8
10
12
14

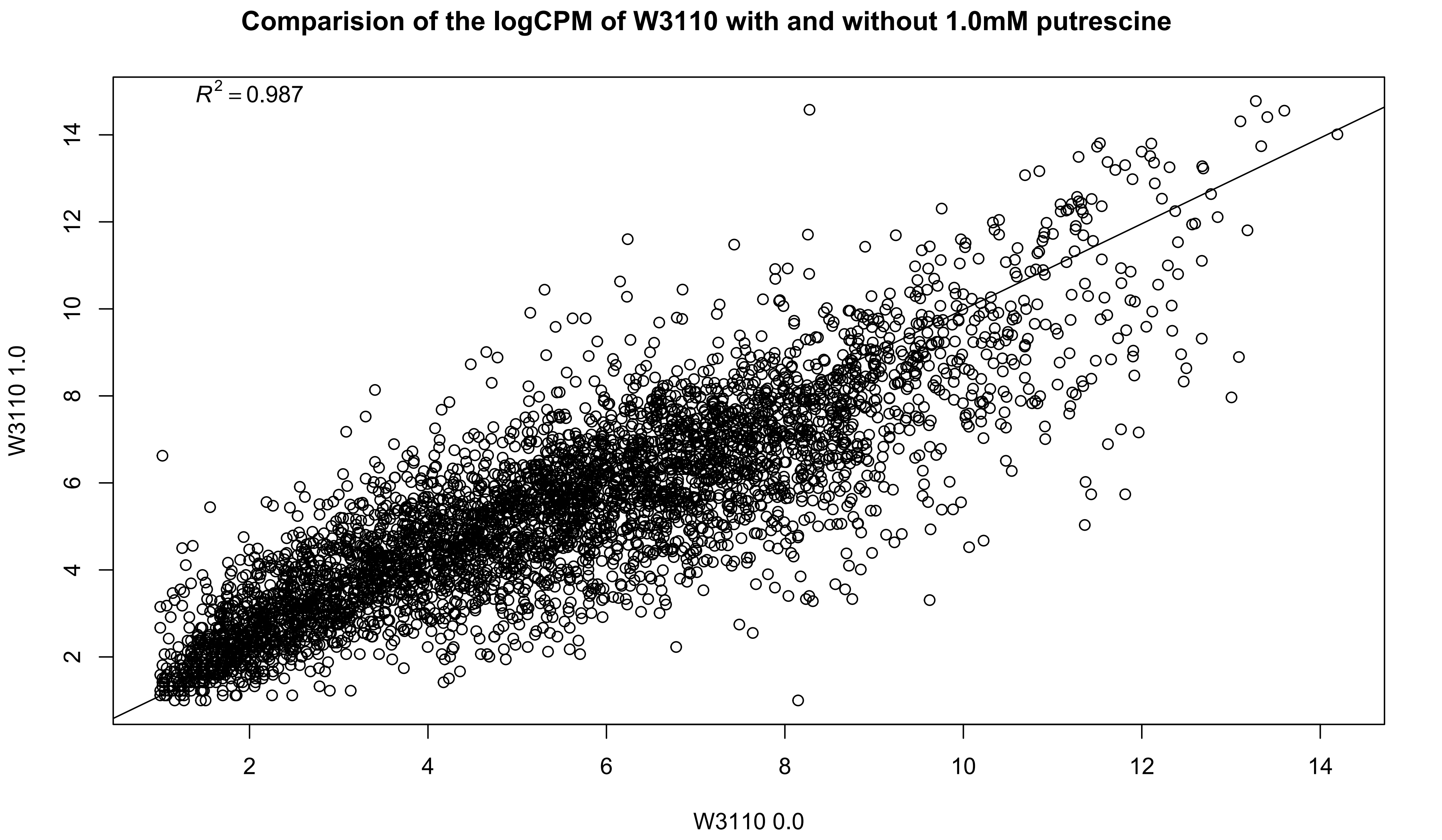

Comparision of the logCPM of W3110 with and without 1.0mM putrescine
$R^2 = 0.987$
W3110 1.0
W3110 0.0
2
4
6
8
10
12
14

Supplementary File 1: Expression graphs comparing the logCPM of the transcriptomes of the average of the three replicates of the W3110 with and without 1 mM putrescine and the *speB* mutant with and without 1 mM putrescine. A regression line was calculated and the correlation between each set of transcriptomes noted on the graph. Higher R^2 values indicate greater similarity between the transcriptomes.
